# Malaria in China, 2011–2015: an observational study

**DOI:** 10.2471/BLT.17.191668

**Published:** 2017-05-26

**Authors:** Shengjie Lai, Zhongjie Li, Nicola A Wardrop, Junling Sun, Michael G Head, Zhuojie Huang, Sheng Zhou, Jianxing Yu, Zike Zhang, Shui-Sen Zhou, Zhigui Xia, Rubo Wang, Bin Zheng, Yao Ruan, Li Zhang, Xiao-Nong Zhou, Andrew J Tatem, Hongjie Yu

**Affiliations:** a School of Public Health, Fudan University, Dongan Road, Xuhui District, Shanghai, 200032, China.; b Division of Infectious Disease, Chinese Center for Disease Control and Prevention, Beijing, China.; c Department of Geography and Environment, University of Southampton, Southampton, England.; d Faculty of Medicine and Global Health Research Institute, University of Southampton, Southampton, England.; e Institute of Pathogen Biology, Chinese Academy of Medical Sciences; f The First Affiliated Hospital College of Medicine, Zhejiang University, Hangzhou, China.; g National Institute of Parasitic Diseases, Chinese Center for Disease Control and Prevention, Shanghai, China.

## Abstract

**Objective:**

To ascertain the trends and burden of malaria in China and the costs of interventions for 2011–2015.

**Methods:**

We analysed the spatiotemporal and demographic features of locally transmitted and imported malaria cases using disaggregated surveillance data on malaria from 2011 to 2015, covering the range of dominant malaria vectors in China. The total and mean costs for malaria elimination were calculated by funding sources, interventions and population at risk.

**Findings:**

A total of 17 745 malaria cases, including 123 deaths (0.7%), were reported in mainland China, with 15 840 (89%) being imported cases, mainly from Africa and south-east Asia. Almost all counties of China (2855/2858) had achieved their elimination goals by 2015, and locally transmitted cases dropped from 1469 cases in 2011 to 43 cases in 2015, mainly occurring in the regions bordering Myanmar where *Anopheles minimus* and *An. dirus* are the dominant vector species. A total of United States dollars (US$) 134.6 million was spent in efforts to eliminate malaria during 2011–2015, with US$ 57.2 million (43%) from the Global Fund to Fight AIDS, Tuberculosis and Malaria and US$ 77.3 million (57%) from the Chinese central government. The mean annual investment (US$ 27 million) per person at risk (574 million) was US$ 0.05 (standard deviation: 0.03).

**Conclusion:**

The locally transmitted malaria burden in China has decreased. The key challenge is to address the remaining local transmission, as well as to reduce imported cases from Africa and south-east Asia. Continued efforts and appropriate levels of investment are needed in the 2016–2020 period to achieve elimination.

## Résumé

**Objectif:**

Déterminer l'évolution et la charge du paludisme en Chine ainsi que les coûts des interventions visant son élimination, pour la période de 2011 à 2015.

**Méthodes:**

Nous avons analysé les caractéristiques spatio-temporelles et démographiques des cas de transmission locale et d'importation du paludisme, en utilisant des données de surveillance désagrégées sur le paludisme couvrant les principaux vecteurs en Chine sur la période de 2011 à 2015. Les coûts totaux et moyens des efforts d'élimination du paludisme ont été calculés pour chaque source de financement, chaque type d'intervention et en fonction de la population exposée.

**Résultats:**

Un total de 17 745 cas de paludisme, dont 123 mortels (0,7%), a été recensé en Chine continentale, avec 15 840 cas importés (89%), essentiellement depuis l'Afrique et l'Asie du Sud-Est. Pratiquement tous les comtés de Chine (2855/2858) ont atteint leurs objectifs vers l’élimination du paludisme en 2015, et les cas de transmission locale ont chuté de 1469 cas en 2011 à 43 cas en 2015, survenus principalement dans les régions bordant le Myanmar, où les espèces *Anophèles minimus* et *An. dirus* sont les principaux vecteurs. Au total, 134,6 millions de dollars des États-Unis (USD) ont été dépensés dans les efforts d'élimination du paludisme entre 2011 et 2015, dont 57,2 millions d'USD (43%) en provenance du Fonds mondial de lutte contre le sida, la tuberculose et le paludisme et 77,3 millions d'USD (57%) en provenance du gouvernement central chinois. L'investissement annuel moyen (27 millions d'USD) par personne exposée (574 millions) a été de 0,05 USD (écart type: 0,03).

**Conclusion:**

La charge des cas de transmission locale du paludisme en Chine a diminué. Le principal défi consiste désormais à lutter contre la transmission locale résiduelle et à réduire les cas importés depuis l'Afrique et l'Asie du Sud-Est. Des efforts continus et des niveaux d'investissement appropriés sont nécessaires sur la période 2016-2020 pour atteindre l'objectif d'élimination du paludisme.

## Resumen

**Objetivo:**

Determinar las tendencias y la carga de paludismo en China y los costes de las intervenciones entre 2011 y 2015.

**Métodos:**

Se analizaron las características espacio-temporales y demográficas de los casos de paludismo importado y transmitido localmente utilizando datos de seguimiento desglosados sobre el paludismo entre 2011 y 2015, y se cubrió la gama de vectores del paludismo dominantes en China. Se calcularon los costes totales y medios para la eliminación del paludismo teniendo en cuenta las fuentes de financiación, las intervenciones y la población en riesgo.

**Resultados:**

En China continental se registró un total de 17 745 casos de paludismo, incluyendo 123 muertes (0,7%), de los cuales 15 840 (89%) fueron casos importados, principalmente de África y el sudeste asiático. Casi todos los condados de China (2 855/2 858) habían logrado sus objetivos de eliminación para 2015, y los casos transmitidos localmente descendieron de 1 469 casos en 2011 a 43 casos en 2015, principalmente en las regiones fronterizas con Myanmar, donde *Anopheles minimus* y *An. dirus* son las especies de vectores dominantes. Se gastó un total de 134,6 millones de dólares estadounidenses (USD) en el esfuerzo por eliminar el paludismo durante el periodo 2011-2015. De este dinero, 57,2 millones de USD (un 43%) provinieron del Fondo Mundial de lucha contra el sida, la tuberculosis y la malaria y 77,3 millones de USD (un 57%) del gobierno central chino. La inversión anual media (27 millones de USD) por persona en riesgo (574 millones) fue de 0,05 USD (desviación estándar: 0,03).

**Conclusión:**

La carga de paludismo transmitido localmente en China ha disminuido. El reto principal es poner remedio a los casos transmitidos localmente que quedan, además de reducir los casos de importación de la infección desde África y el sudeste asiático. Durante el periodo 2016-2020, se necesitan esfuerzos continuos y niveles de inversión adecuados para lograr su eliminación.

## ملخص

الغرض ****الوقوف على الاتجاهات السائدة وأعباء الملاريا في الصين وتكاليف التدخلات للفترة ما بين عامي 2011 و2015.

الطريقة ****حللنا السمات المكانية والزمانية والديمغرافية لحالات الملاريا المنقولة محليًا والواردة باستخدام بيانات مراقبة مفصلة عن الملاريا في الفترة ما بين عامي 2011 و2015، والتي تغطي مجموعة ناقلات الملاريا السائدة في الصين. وقد تم حساب التكاليف الإجمالية والمتوسطة للتخلص من الملاريا على أساس مصادر التمويل والتدخلات والقطاعات السكانية المعرضة للخطر.

النتائج ****وقد تم الإبلاغ عن وجود 17,745 حالة من حالات الملاريا، بما في ذلك 123 حالة وفاة (0.7‏%)، في البر الرئيسي للصين، مع وجود 15,840 حالة واردة (89‏%)، معظمها من أفريقيا وجنوب شرق آسيا. حققت جميع مقاطعات الصين تقريبًا (2855/2858) أهداف القضاء عليها بحلول عام 2015، وانخفضت الحالات المنقولة محليًا من 1469 حالة في عام 2011 إلى 43 حالة في عام 2015، والتي ظهرت أساسًا في المناطق المتاخمة لميانمار حيث تكون كلاً من الأنوفيلة الصغيرة والأنوفيلة الفظيعة كأنواع مسيطرة ناقلة للأمراض. وتم إنفاق ما يصل إلى 134.6 مليون دولار أمريكي في إطار الجهود المبذولة للقضاء على الملاريا خلال الفترة ما بين عامي 2011 و2015، حيث تم الحصول على 57.2 مليون دولار أمريكي (43‏%) من الصندوق العالمي لمحاربة الإيدز والسل والملاريا، و77.3 مليون دولار أمريكي (‏57%) من الحكومة المركزية الصينية. وبلغ متوسط الاستثمار السنوي (27 مليون دولار أمريكي) للشخص المعرض للخطر (574 مليون شخص) 0.05 دولار أمريكي (الانحراف المعياري: 0.03).

الاستنتاج ****انخفض عبء الملاريا المنقولة محليًا في الصين. ويتمثل التحدي الرئيسي في معالجة النقل المحلي المتبقي، بالإضافة إلى الحد من الحالات الواردة من أفريقيا وجنوب شرق آسيا. ويلزم بذل جهود متواصلة ومستويات مناسبة من الاستثمار في الفترة ما بين عامي 2016 و2020 لتحقيق القضاء الكامل عليها.

## 摘要

**目的:**

旨在明确 2011-2015 年间中国的疟疾流行趋势和压力及其干预措施成本。

**方法:**

我们使用 2011 至 2015 年间的分散型疟疾监测数据(涵盖中国的显性疟疾媒介的范围)分析了本地传播型及输入型疟疾病例的时空特征和人口特征。 消除疟疾的总成本及平均成本按资金来源、干预措施及危险人群数量进行计算。

**结果:**

中国大陆共报告了 17 745 件疟疾病例,其中包括 123 件 (0.7%) 死亡案例,15 840 件 (89%) 输入型病例。输入来源主要为非洲及东南亚。 中国几乎所有区县 (2855/2858) 已在 2015 年实现了其消灭疟疾的目标,本地传播型病例已由 2011 年的 1469 例下降至 2015 年的 43 例,主要发病区位于缅甸边境地带,该地区主要的媒介种类是*微小按蚊*和*大劣按蚊*。 2011-2015 年间共计支出 13,460 万美元 (US$) 用于消除疟疾,其中 5,720 万 (43%) 美元来源于抗击艾滋病、结核病和疟疾全球基金，7,730 万 (57%) 美元来源于中国中央政府。 每位危险人群(共 57,400 万人)的平均年投资(2,700 万美元)为 0.05 美元(标准差为: 0.03)。

**结论:**

中国的本地传播型疟疾疫情已经减轻。 应对剩余的本地传播以及减少来自非洲及东南亚地区的输入型病例是主要挑战。 2016-2020 年期间,中国仍需继续努力并进行适当投资以实现消除疟疾的目标。****

## Резюме

**Цель:**

Выяснить тенденции и нагрузку в отношении малярии в Китае, а также определить расходы на проведение необходимых мероприятий в период 2011–2015 гг.

**Методы:**

Мы провели анализ пространственно-временных и демографических особенностей случаев местнотрансмиссивной и завезенной малярии с использованием дезагрегированных данных эпиднадзора за малярией в период с 2011 по 2015 год, охватывающих диапазон доминирующих векторов малярии в Китае. Общие и средние затраты на ликвидацию малярии рассчитывались с учетом источников финансирования, необходимых мероприятий и групп населения, подверженных риску.

**Результаты:**

Всего в континентальном Китае было зарегистрировано 17 745 случаев малярии, в том числе 123 случая со смертельным исходом (0,7%), из которых 15 840 случаев (89%) были завезены, главным образом из Африки и Юго-Восточной Азии. Почти все уезды Китая (2855 из 2858) достигли своих целей по ликвидации заболевания к 2015 году, а число местнотрансмиссивных случаев сократилось с 1469 случаев в 2011 году до 43 случаев в 2015 году, главным образом в регионах, граничащих с Мьянмой, где *Anopheles minimus* и *An. dirus* являются доминирующими видами-переносчиками. В рамках усилий по ликвидации малярии в 2011–2015 гг. было израсходовано 134,6 млн долл. США, из которых 57,2 млн долл. США (43%) были выделены из Глобального фонда *для борьбы со СПИДом*, *туберкулезом и малярией*******и 77,3 млн долл. США (57%) были инвестированы правительством Китая. Средние ежегодные инвестиции (27 млн долл. США) из расчета на одного человека, подверженного риску заразиться малярией (574 млн), составили 0,05 долл. США (стандартное отклонение: 0,03).

**Вывод:**

Местнотрансмиссивное бремя малярии в Китае снизилось. Основная задача состоит в том, чтобы решить сохраняющуюся проблему местной передачи инфекции, а также сократить случаи завоза инфекции из Африки и Юго-Восточной Азии. Для завершения ликвидации в 2016–2020 гг. необходимы дальнейшие усилия и соответствующие уровни инвестиций.

## Introduction

Malaria remains a public health issue, with an estimated 214 million cases and 438 000 deaths globally in 2015.^1^^,^^2^ Historically, malaria has been widespread in China, with 24 malaria-endemic provinces and over 24 million cases being reported in the early 1970s. *Plasmodium vivax* and *P. falciparum* are the main parasite species responsible.^3^^,^^4^ After control efforts were intensified in China in 2007, the incidence of malaria was substantially reduced in the provinces with malaria transmission, with 95% of these counties (2345/2469) having an estimated incidence below 1 per 10 000 persons in 2009.^5^ The Chinese government launched a national malaria elimination programme in May 2010, aimed at reducing the number of locally transmitted malaria cases across most of China to zero by 2015 (except in some border areas of Yunnan province where the goal is elimination by 2017), and achieving World Health Organization (WHO) certification of malaria elimination for China by 2020.^3^^,^^6^ Comprehensive intervention policies and strategies have been adopted,^7^^,^^8^ and in 2014 indigenous malaria infections were only found in Yunnan and Tibet provinces.^9^

Both international and domestic funds have been used to implement the national malaria elimination programme to achieve the goal of malaria elimination. The Global Fund to Fight AIDS, Tuberculosis and Malaria has supported China, with approximately United States dollars (US$) 113 million, to progress from control to elimination between 2003 and 2012.^9^^,^^10^ Hence the coverage of Global Fund-supported projects expanded from 47 high malaria-burden counties (within 10 provinces) in 2003 to 762 high and lower malaria-burden counties (within 20 provinces) in 2010.^9^ The Global Fund accounted for all documented operational malaria funding in China between 2005 and 2010,^11^ and the national strategy application project from the Global Fund has been specific for malaria elimination in China since 2010. However, changes to eligibility criteria in November 2011 meant that China was no longer eligible for grant renewals, due to its categorization as an upper-middle-income country and the malaria burden being sufficiently low.^9^^,^^10^ The national strategy application was closed ahead of schedule on 30 June 2012, and the Chinese central government has since been committed to covering the investment gaps.^9^

Few comprehensive analyses of the changing epidemiology of malaria in China have been done. The achievement of the national malaria elimination programme by 2015, the challenges for the halfway point goals and the evidence in favour of these actions has been more descriptive than quantitative.^9^^,^^12^^–^^15^ Both donors and policy-makers should ideally have information about the costs and benefits of interventions.^16^^–^^18^ A robust epidemiological and cost analysis is important to support the design and update of national strategies and future needs for malaria elimination.^17^^–^^19^ We conducted an observational analysis to determine (i) the epidemiological trends and burden of malaria; (ii) the areas and populations with residual transmission; and (iii) the costs of interventions from different donors for malaria elimination from 2011 to 2015. This work identifies the achievements and challenges and thereby helps to plan resource allocation for the second half (2016–2020) of the elimination plan and the ultimate goals of the national malaria elimination programme in China.

## Methods

### Data sources

We obtained data on individual malaria cases, including clinically diagnosed and laboratory-confirmed cases reported in all 31 provinces of mainland China during 2011–2015, from the Chinese malaria enhanced surveillance information system. This system was developed as a part of the national malaria elimination programme to actively collect demographic and epidemiological information, using the unified form for case investigation required by the Chinese technical scheme of malaria elimination.^7^^,^^20^ Laboratory-confirmed malaria cases refer to patients with a positive result from one type of laboratory test.^21^ Rapid diagnostic tests were the primary diagnostic tools in the remote villages, townships and counties. Microscopy was used in county, prefectural and provincial levels as the gold standard method for case verification. Polymerase chain reaction was mainly used for case verification at provincial levels because of its higher sensitivity than microscopy and rapid diagnostic tests. Clinically diagnosed cases were defined as patients with malaria-like symptoms but no parasites detected in blood examination. Imported cases were malaria patients who had travelled to any malaria-endemic areas outside China within the month before illness onset; the last country visited was taken as the potential origin of infection. Locally transmitted cases were patients who had contracted malaria within China.^7^

We extracted data on the costs of malaria control and the estimated annual population at risk in 2011–2015 from the WHO annual world malaria reports for 2012–2016,^1^^,^^22^^–^^25^ the annual report of malaria elimination in China, the national programme office for malaria of the Global Fund in China, and information publicly available through the Global Fund website.^10^ This study included the costs from the Global Fund (2011–2012) and Chinese central government (2011–2015). Other sources of international malaria funding (e.g. the President’s Malaria Initiative, the United Nations International Children’s Emergency Fund and The World Bank) were checked but excluded because no funding for malaria was allocated to China from these sources in 2011–2015. The costs incurred by the governments at sub-national levels are also not included here because the Chinese central government plays a major role in domestic funding to the national malaria elimination programme. From the world malaria reports^1^^,^^22^^–^^25^ and the China annual reports of malaria elimination,^26^^–^^30^ we also collected data on actions supported using these funds: the number of long-lasting insecticidal nets and insecticide-treated nets distributed; the number of people protected by indoor residual spraying; and the number of blood samples collected and tested for malaria. All the funds documented in Chinese yuan were converted into US$ (the conversion rates were US$ 100 to Chinese Yuan: 645.88 in 2011; 631.25 in 2012; 619.32 in 2013; 614.28 in 2014; and 622.84 in 2015),^31^ were adjusted for the annual average inflation rate in China, to measure funding or spending trends in real terms.

The geographical distributions of dominant *Anopheles* species vectors of human malaria in China were obtained from the Malaria Atlas project^32^ to define high-risk areas for malaria residual transmission. The population data at national and sub-national level for each year were obtained from the national statistical bureau of China,^31^ to estimate the incidence rate and population living in counties with malaria transmission by different dominant *Anopheles* mosquitoes.

### Data analyses

For this analysis we included all cases reported in all 2858 counties of 333 prefectures in 31 provinces of mainland China, with illness onset from 1 January 2011 to 31 December 2015. We summarized the epidemiological characteristics of malaria cases. We estimated the incidence for each year at national and county levels, and calculated the malaria case-fatality rate (number of deaths divided by number of probable and confirmed cases), both overall and stratified by locally transmitted and imported cases. All counties in mainland China have been classified into four categories with different goals for malaria elimination in the national malaria elimination programme (Table 1). We defined the achievement of the national malaria elimination programme for 2011–2015 by comparing the incidence of malaria with the midway goals of the four categories of counties by 2015. The population living in the counties with locally transmitted *P. falciparum* and *P. vivax* each year were stratified by the different dominant *Anopheles* vectors.

**Table 1:** Four categories of counties in mainland China and their goals and achievements for malaria elimination

Category definition	No. (%) of counties (*n* = 2858^a^)	Goals by 2015	Goals in 2016–2020	Achievement of goals by 2015
Local infections detected in 3 consecutive years and annual incidence ≥ 1 per 10 000 persons for each year	75 (3)	Counties in border areas of Yunnan: annual incidence < 1 per 10 000 persons	Counties in border areas of Yunnan: no local infections detected by 2017; malaria elimination by 2020	Yes. Annual incidence in each county was < 1 cases per 10 000 persons
		Other counties: no local infections detected by 2015	Other counties: malaria elimination by 2018	Partly. Motuo county in Tibet (bordering with India) and Sanya city in Hainan in the tropics reported locally transmitted cases in 2015. Motuo county had ≥ 1 case per 10 000 persons for each year in 2011–2015
Local infections detected in the last 3 years and an annual incidence < 1 per 10 000 persons in 1 of the 3 years	687 (24)	No local infections detected by 2015	Malaria elimination by 2018	Partly. Donggang city in Liaoning province (bordering with the Democratic People's Republic of Korea) reported locally transmitted cases in 2015
No local infections reported in the last 3 years	1432 (50)	Malaria elimination by 2015	Maintaining malaria-free status	Yes. Passed the subnational malaria elimination assessment
Non-malaria-endemic area	664 (23)	Maintaining malaria-free status	Maintaining malaria-free status	Yes. Maintained malaria-free status

^a^ Only the counties of 31 provinces in mainland China are included in the national malaria elimination programme.

Note: The counties are categorized by the malaria incidence data reported in mainland China from 2006 to 2008, obtained from the Action Plan of China Malaria Elimination (2010–2020) and the National Notifiable Infectious Disease Reporting Information System in China.^3^^,^^5^

We calculated the values of funds disbursed for malaria elimination by the Global Fund and the Chinese central government in 2011–2015. The costs of different interventions and management (e.g. insecticidal nets, diagnostic testing, insecticide and spraying materials, antimalarial medicines, monitoring and evaluation, human resources and technical assistance, management, and other costs) were summarized for each year and stratified by sources of funding. We estimated the coverage of nets (long-lasting insecticidal nets and insecticide-treated nets) and indoor residual spraying, using the corresponding at-risk population in China. The proportion of positive cases was calculated by dividing the total number of laboratory-confirmed malaria cases by the number of blood samples tested, multiplied by 100 (and expressed as a percentage).

We used *R* statistical software version 3.3.1 (R Foundation for Statistical Computing, Vienna, Austria) for statistical analyses.

### Ethical approval

The National Health and Family Planning Commission of China determined that the collection of malaria case reports was part of continuing public health surveillance of a notifiable infectious disease and was exempt from institutional review board assessment. Ethical clearance for collecting and using second-hand data was also granted by the institutional review board of the University of Southampton, England (No. 18152). All data were supplied and analysed in an anonymous format, without access to personal identifying information.

## Results

**Fig. 1:**
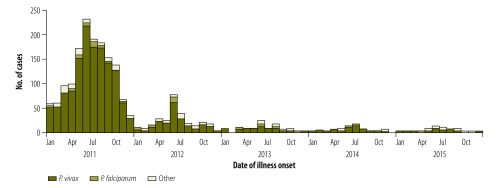
Epidemic curves of locally transmitted cases of malaria in mainland China, 2011–2015

Notes: Locally transmitted cases (*n* = 1905) included 1708 cases of *Plasmodium vivax* infection, 92 *P. falciparum*, 4 *P. malariae*, 1 *P. ovale*, 5 mixed infections and 95 untyped. The total annual population of China was: 1347 million (2011); 1354 million (2012); 1361 million (2013); 1368 million (2014); and 1375 million (2015).^31^

From 2011 to 2015, a total of 17 745 malaria cases, including 123 deaths (0.7%), were reported in mainland China, of which 1905 (11%) were locally transmitted (Fig. 1). The number of locally transmitted malaria cases dropped from 1469 in the total population of about 1.3 billion in 2011 (1.1 cases per 1 000 000 persons) to 43 in 1.4 billion in 2015 (0.03 cases per 1 000 000 persons). Most locally transmitted cases over this period (1708; 90%) were infected with *P. vivax*.

Data sources: The number of malaria cases were obtained from the Chinese malaria enhanced surveillance information system.^7^^,^^20^

Compared with the goals set for different counties in the national malaria elimination programme by 2015, almost all counties (2855/2858) had achieved their goals by 2015. All 25 counties in the border areas of Yunnan had an annual incidence below the target of one case per 10 000 persons since 2013. However, Motuo county in Tibet, Sanya city in Hainan Island and Donggang city in Liaoning province failed to achieve their goal (reducing locally transmitted cases to zero) by 2015 (Table 1).

The residual transmission by 2015 might reflect the spatial variability and complexity of *Anopheles* vectors in China. Among the counties with only *An. sinensis* and/or *An. lesteri* as dominant vectors, the number of *P. vivax* and *P. falciparum* cases decreased substantially, with only one county reporting the occurrence of locally transmitted *P. vivax* in 2015 (Table 2). However, among the counties with other dominant vectors (e.g. *An. minimus, sensu lato (s.l.)*, *An. dirus s.l.*, *An. stephensis*, and *An. maculatus*), there were still more than 10 counties (with a combined population of about 3 766 000) reporting locally transmitted *P. vivax* annually in 2013–2015, and two counties (with a combined population of 569 000) reporting locally transmitted *P. falciparum* in 2015.

**Table 2:** Trends in locally transmitted *Plasmodium vivax* and *P. falciparum* malaria infections in mainland China, 2011–2015

Variable	Year				
	2011	2012	2013	2014	2015
**Total population, thousands**	1 347 350	1 354 040	1 360 720	1 367 820	1 374 620
***P. vivax and P. falciparum* malaria**					
Total no. of cases	1396	231	78	59	36
No. of cases per 1 000 000 persons	1.04	0.17	0.06	0.04	0.03
No. of counties affected	183	50	21	10	11
Population of counties, thousands	104 499	25 940	9 202	1 872	3 945
***P. vivax* malaria**					
Total no. of cases	1344	212	65	53	34
No. of cases per 1 000 000 persons	1.00	0.16	0.05	0.04	0.02
No. of counties affected (% of total)					
Total	182 (100)	50 (100)	18 (100)	10 (100)	10 (100)
Only *Anopheles sinensis* and/or *An. lesteri* mosquitoes	119 (65)	24 (48)	5 (28)	0 (0)	1 (10)
Other *Anopheles* mosquitoes^a^	63 (35)	26 (52)	13 (72)	10 (100)	9 (90)
Population in counties affected, thousands (% of total)					
Total	104 242 (100)	25 940 (100)	7 622 (100)	1 872 (100)	3 766 (100)
Only *An. sinensis* and/or *An. lesteri* mosquitoes	84 376 (81)	18 199 (70)	3 937 (52)	0 (0)	627 (17)
Other *Anopheles* mosquitoes^a^	19 866 (19)	7741 (30)	3 685 (48)	1 872 (100)	3 139 (83)
***P. falciparum* malaria**					
Total no. of cases	52	19	13	6	2
No. of cases per 1 000 000 persons	0.04	0.01	0.01	0.00	0.00
No. of counties affected (% of total)					
Total	17 (100)	9 (100)	6 (100)	2 (100)	2 (100)
Only *An. sinensis* and/or *An. lesteri* mosquitoes	2 (12)	0 (0)	3 (50)	0 (0)	0 (0)
Other *Anopheles* mosquitoes^a^	15 (88)	9 (100)	3 (50)	2 (100)	2 (100)
Population in counties affected, thousands (% of total)					
Total	4 391 (100)	2 941(100)	2 246 (100)	484 (100)	569 (100)
Only *An. sinensis* and/or *An. lesteri* mosquitoes	362 (8)	0 (0)	1 581 (70)	0 (0)	0 (0)
Other *Anopheles* mosquitoes^a^	4 029 (92)	2 941 (100)	665 (30)	484 (100)	569 (100)

^a^ Other *Anopheles* mosquitoes includes *An. minimus s.l.*, *An. dirus s.l.*, *An. stephensis* and *An. maculatus*.

Notes: 11 counties in 5 provinces (Yunnan, Tibet, Hainan, Guangxi and Liaoning) reported locally transmitted cases in 2015.

Data sources: The malaria data were obtained from the Chinese malaria enhanced surveillance information system.^7^^,^^20^ The geographical distribution of dominant *Anopheles* vectors of human malaria in China was obtained from the Malaria Atlas project.^32^ The population data at national and sub-national level for each year were obtained from the national statistical bureau of China.^31^

**Fig. 2:**
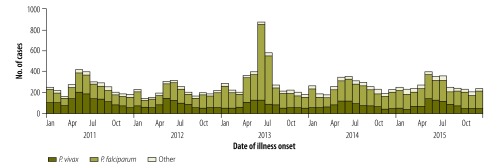
Epidemic curves of imported cases of malaria in mainland China, 2011–2015

Notes: Imported cases (*n* = 15 840) included 9754 cases of *Plasmodium falciparum* infection, 4882 *P. vivax*, 524 *P. ovale*, 188 *P. malariae*, 202 mixed infections and 290 untyped. The total annual population of China was: 1347 million (2011); 1354 million (2012); 1361 million (2013); 1368 million (2014); and 1375 million (2015).^31^

A total of 15 840 (89%) imported malaria cases were reported from 2011 to 2015 (Fig. 2). All 31 provinces reported cases, with a median of 3091 cases per year (interquartile range, IQR: 3049‒3221 cases). The imported cases originated from 69 countries (44 in Africa, 18 in south-east Asia and seven in other regions). Most imported cases were among males (14 972; 95%) and Chinese nationality migrant workers (14 849; 94%). The median stay was longer in Africa (320 days; IQR: 171–515) than in south-east Asia (120 days; IQR: 59–229). 

Data sources: The number of malaria cases were obtained from the Chinese malaria enhanced surveillance information system.^7^^,^^20^

Most cases imported from Africa (8756/10 949; 80%) were infected with *P. falciparum*, whereas a high proportion (3362/4340; 78%) of cases from south-east Asia were due to *P. vivax*. The majority of cases from south-east Asia were imported to Yunnan province (3082; 71%), whereas cases from Africa were mostly imported to Guangxi (1834; 17%), Jiangsu (1603; 15%) and Sichuan (884; 8%) provinces. For *P. vivax*, 1536 counties (54% of all 2858 counties) only reported imported cases, six counties (0.2%) only reported locally transmitted cases and 18 counties (0.6%) had both. For *P. falciparum*, 857 counties (30%) only reported imported cases, 90 counties (3%) only reported locally transmitted cases and 103 counties (4%) had both.

China spent a total of US$ 134.6 million on malaria elimination efforts during 2011–2015, including US$ 57.2 million (43%) from the Global Fund in 2011–2012 and US$ 77.3 million (57%) from the central government of China in 2011–2015 (Table 3). The value of funding varied each year, with the highest (US$ 51.5 million) provided in 2012, and subsequent reductions between 2013 and 2015 after the Global Fund ceased providing funds. The level of funding from the Chinese central government increased to fill the gap during the transition of funders, but the annual values were still lower than that previously provided by the Global Fund. The mean annual investment from 2011 to 2015 was US$ 27 million for about 574 million people at risk (i.e. living in counties with malaria transmission), or US$ 0.05 per person at risk (standard deviation, SD: 0.03). 

**Table 3:** Interventions and costs for malaria elimination in mainland China, 2011–2015

Variable	Year				
	2011	2012	2013	2014	2015
**Population, thousands (% of total)**					
Total	1 347 350	1 354 040	1 360 720	1 367 820	1 374 620
At risk of malaria^a^	563 574 (42)	575 911 (42)	579 467 (42)	575 985 (42)	575 985 (42)^b^
At high risk of malaria^a^	192 (0.01)	196 (0.01)	197 (0.01)	196 (0.01)	196 (0.01)
**Funding, US$ millions (% of total)**					
Total	31.5 (100)	51.5 (100)	16.0 (100)	19.4 (100)	16.2 (100)
The Global Fund	24.4 (77)	32.8 (64)	0.0 (0)	0.0 (0)	0.0 (0)
Central Government of China	7.1 (23)	18.6 (36)	16.0 (100)	19.4 (100)	16.2 (100)
**Spending per person at risk, US$**	0.06	0.09	0.03	0.04	0.03
**Spending on interventions, US$ millions (% of total)^c^**					
Total	24.4 (100)	N/A	16.0 (100)	19.4 (100)	16.2 (100)
Insecticide and spraying materials	0.5 (2)	N/A	1.1 (7)	0.8 (4)	0.7 (4)
Insecticide-treated nets and long-lasting insecticidal nets	0.4 (1)	N/A	1.4 (9)	1.1 (6)	0.9 (6)
Diagnostic testing	0.7 (3)	N/A	13.3 (83)	8.9 (46)	7.5 (46)
Antimalarial medicines	0.0 (0)	N/A	0.2 (1)	0.2 (1)	0.2 (1)
Monitoring and evaluation	2.7 (11)	N/A	0.0 (0)	0.0 (0)	0.0 (0)
Human resources and technical assistance	6.3 (26)	N/A	0.0 (0)	0.3 (2)	0.3 (2)
Management and other costs	13.8 (57)	N/A	0 (0)	8.1 (42)	6.6 (41)
**Nets coverage, no. of nets purchased**					
Total	656 674	509 490	58 874	19 899	29 611
Long-lasting insecticidal nets	149 394	251 555	58 874	19 899	29 611
Insecticide-treated nets	507 280	257 935	0	0	0
**Indoor residual spraying coverage, no. of people protected**	1 043 963	1 092 158	447 639	504 936	1 697 188
**Laboratory-confirmed malaria, no. of blood samples**					
Total collected	9 189 270	6 918 657	5 554 960	4 403 633	4 052 588
Positive (% of total)^d^	3629 (0.04)	2633 (0.04)	4029 (0.07)	3065 (0.07)	3223 (0.08)
Positive, by species (% of positive samples)					
*P. falciparum*	1467 (40)	1460 (55)	2892 (72)	1879 (61)	1977 (61)
*P. vivax*	2087 (58)	1068 (41)	915 (23)	919 (30)	910 (28)
Other^e^	75 (2)	105 (4)	222 (6)	267 (9)	336 (11)

N/A: data not available; The Global Fund: The Global Fund to Fight AIDS, Tuberculosis and Malaria; US$: United States dollars.

^a^ Using the estimates of population at risk in 2014 from the world malaria report 2015.^1^

^b^ Risk areas (counties) were those with malaria transmission. High-risk areas (counties) were those with > 1 case per 1000 persons.

^c^ Expenditure by interventions in 2011 only included the costs incurred by the Global Fund. Cost calculations did not include: salaries of department of health staff at county level or above; direct costs of eliminating malaria incurred by the governments at sub-national levels; costs of treatment of malaria provided by physicians; or expenditure on malaria treatment by patients. All the funds documented in Chinese yuan were converted into US$ using the average exchange rate from the year of the award of funding, and the values were adjusted for the annual average inflation rate in China (2.65% in 2012, 2.62% in 2013, 1.99% in 2014 and 1.44% in 2015) through comparison to 2011.

^d^ Malaria cases were confirmed by diagnostic tests of microscopy, rapid diagnostic tests or polymerase chain reaction tests.

^e^ Other included *P. ovale*, *P. malariae*, and mixed infections.

Notes: The conversion rates were US$ 100 to Chinese Yuan: 645.88 in 2011; 631.25 in 2012; 619.32 in 2013; 614.28 in 2014; and 622.84 in 2015.^31^

Data sources: The data of malaria cases were obtained from the Chinese malaria enhanced surveillance information system.^7^^,^^20^ The estimated annual population at risk in 2011–2015 were extracted from the WHO annual world malaria reports for 2012–2016,^1^^,^^22^^-^^25^ and the population data at national level for each year were obtained from the national statistical bureau of China.^31^ The costs of malaria control in 2011–2015 were extracted from the WHO annual world malaria reports for 2012–2016,^22^^-^^25^ the China annual report of malaria elimination, the national programme office for malaria of the Global Fund in China, and information publicly available through the Global Fund website.^10^

The expenditure by intervention varied between international and domestic funding (Table 3). In 2011, the expenditure on management and other costs (e.g. vehicle, small refrigerators and computers) accounted for US$ 13.8 million (57%) of the US$ 24.4 million from the Global Fund. The next highest cost was human resources and technical assistance (US$ 6.3 million; 26%) for providing township hospitals and village clinics with incentives to improve case management and reporting. However, over 2013‒2015 the total financing for interventions from the Chinese central government (US$ 51.6 million) was predominantly allocated for diagnostic testing (US$ 29.7 million; 58%) and management and other costs (US$ 14.7 million; 28%). The costs of insecticide-treated nets and long-lasting insecticidal nets, insecticide and spraying materials and antimalarial medicines accounted for small proportions of both international (US$ 0.9 million; 4%) and domestic (US$ 6.6 million; 13%) funding. 

A total of 1 274 548 nets were purchased over 2011‒2015: 509 333 (40%) long-lasting insecticidal nets and 765 215 (60%) insecticide-treated nets. There were decreases in the annual numbers of nets purchased each year from 2011 to 2014, and in the high-risk populations (i.e. living in counties with > one case per 1000 persons) covered by indoor residual spraying from 2011 to 2015 (Table 3). Of the total of 30 119 108 blood samples collected for testing 16 579 (0.06%) were positive for malaria parasites (Table 3).

## Discussion

The incidence of locally transmitted malaria has decreased in mainland China following the first five years of elimination efforts which began in May 2010. The geographical range of endemic areas with *P. falciparum* and *P. vivax* transmission has shrunk, with most counties having achieved their national malaria elimination programme goals by 2015. Malaria is on the verge of elimination in central China. This reduction corresponded with the implementation of the national malaria elimination programme and continuous investments from international and domestic funders to support diagnosis and treatment, indoor residual spraying and the distribution of insecticidal nets.^11^^,^^33^ This success could also be attributed, at least in part, to robust surveillance systems that rapidly detected and responded to individual cases.^8^ This study also suggests that the greatest threats to successful elimination efforts in China are residual malaria transmission in the regions with dominant vectors of *An. minimus s.l.* and *An. dirus s.l*.

In areas where malaria transmission has been interrupted, the challenge is to maintain malaria-free status and prevent reintroduction. In contrast, in areas with ongoing local transmission two main challenges exist. First is the higher malaria burden and lack of health care and malaria control services in the malaria-endemic areas of Myanmar and India which border China. Second is the importation of cases from mobile and migrant populations.^34^ A high incidence of clinical malaria has been reported from the villages in Yunnan along the border with Myanmar, and the risk increases closer to the international border.^32^^,^^35^^–^^37^ Malaria parasites could be carried across the borders by infected mosquitoes due to the very close proximity of villages along the border on both sides.^35^^,^^36^ Additionally, malaria importation from beyond neighbouring countries in Africa and south-east Asia also remains a challenge,^38^ because only a few countries in these regions are expected to eliminate malaria by 2020. Therefore, addressing cross-border malaria carried by travellers, especially Chinese migrant workers, to and from Africa and nearby countries in south-east Asia is crucial to eliminate malaria and maintain the gains that have been achieved by China so far.^14^^,^^39^^–^^44^

The cost per person at risk in China was low compared with other countries.^17^^,^^23^ Among 87 malaria-endemic countries that received financial support from international donors to control malaria from 2008 to 2012, China (with more than 56 million people living in endemic districts) ranked second in terms of the size of population at risk of malaria, but 82nd in terms of the amount of international funding invested per person.^17^^,^^23^

For the second half of the elimination programme and post-elimination era in China, the central and local governments will continue to fund malaria elimination activities and ensure that the universal coverage of interventions is maintained. Resurgence of malaria may occur if control and surveillance measures are scaled back too early following elimination; consistent financing is necessary to avoid this.^5^^,^^45^^,^^46^ Malaria elimination in China may be currently underfunded relative to the frequency of parasite importation and the size of the population living in areas at risk of malaria. Increased funding could be crucial for elimination efforts.

This study had some limitations. First, it is possible that not all improvements in the malaria situation were attributable to the elimination activities. For example, it is known that socioeconomic development can be associated with reduced malaria risk in urban areas,^47^ and China has undergone substantial socioeconomic growth and urbanization since the 1980s.^48^ These changes could have contributed to a decrease in malaria prevalence, irrespective of malaria control and elimination activities. Second, the number of malaria cases identified in the present study might be an underestimate if some people did not seek treatment. Also, imported cases of malaria may have been misdiagnosed in malaria-free or hard-to-reach areas, even though the individual case-based malaria surveillance system in China operated well during the malaria elimination stage.^20^ Third, the cost calculations did not include funding from governments at national and sub-national levels to support the salaries of health department staff at county level or above. These staff members were responsible for most of the malaria elimination activities (e.g. surveillance, data collection, vector control and diagnosis). We also did not include the direct costs of eliminating malaria incurred by the governments at sub-national levels. Because of difficulties in obtaining adequate data, we did not include the costs of malaria treatment provided by physicians and the costs to patients.

The results of this study show that the malaria burden in China fell markedly during the study period, with substantial financial support from international and domestic funds. Elimination of malaria is a realistic aim, and the benefits are not only local, but also international if elimination in China acts to reduce or delay the spread of artemisinin resistance from the Mekong region. However, the foreseeable challenges presented here need national attention to achieve the goal of malaria elimination in China by 2020. Investment needs to be maintained and ideally increased to target resources towards the remaining high-burden and high-importation regions. Strong surveillance and response systems need to be maintained to monitor residual transmission in endemic areas. Robust spatiotemporal models linking to disease data and different environmental factors are also needed. These would act as early-warning tools to monitor the risk of importation and predict the onward transmission potential in importation risk areas. This will ensure that elimination is sustained and will form a cornerstone of post-2015 elimination strategies in China.^6^
